# COVID-19: Nurses have responded, now it is time to support them as we move forward

**DOI:** 10.1177/0840470420953297

**Published:** 2020-08-20

**Authors:** Rhonda Collins

**Affiliations:** 1 Chief Nursing Officer, Vocera, San Jose, CA, USA.

## Abstract

The inspiration for The American Nurse Project, Dr. Rhonda Collins, DNP, RN, FAAN, is Chief Nursing Officer for Vocera Communications. Every year around Nurses Week, Dr. Collins publishes a report examining important issues that impact the nursing profession worldwide. Her 2020 CNO report looks at many of the challenges impacting nurses before, during, and after COVID-19—highlighting the mental anguish and physical burdens that COVID-19 has placed on nurses and other frontline healthcare workers as they put themselves in harm’s way to protect others. Dr. Collins examines the foundation of cognitive science and outlines a three-point strategy to guide hospital and nurse leaders moving forward as they strive to support staff members: lightening clinicians’ cognitive load by addressing the difference between documentation and communication, relieving the burden of adapting to multiple systems by giving clinicians control over how they communicate, and providing clinicians with clear, contextual, just-in-time information—using software to enhance workflow, not distract from it. During these unprecedented times, health leaders can honour nurses by providing them with the tools to help strengthen resiliency and healing from this crisis.

## Introduction

Worldwide, there have been over 21 million confirmed cases of COVID-19, and sadly, more than 775,000 people have lost their lives (https://coronavirus.jhu.edu/map.html). The pandemic has had a huge impact on healthcare workers across the globe, and as surges peak and second waves are anticipated, it will continue to do so for the foreseeable future. The mental and physical burden nurses must carry in this time of need is life altering—highlighting the issue of cognitive overload more than ever before. I find myself overwhelmed with a sense of urgency to DO something. Every day, I contemplate the toll on nurses and clinicians who are working endless hours, putting themselves in harm’s way to protect us all.

Each May, I write a CNO Perspective report on a topic that is top of mind for nurse leaders. Last year, I wrote about reducing the cognitive load for nurses and other healthcare professionals. I’ve spent the past year speaking and publishing on this topic, often by invitation.

The response I’ve continued to receive from nurse leaders tells me the theme of reducing cognitive load remains important and timely. My plan for this year’s report has been to go more deeply into how to address many of the challenges nurse leaders have expressed to me.

Since watching the toll COVID-19 has taken on healthcare workers, I now ask myself: how can I possibly talk about anything but the pandemic impacting our world? What are our next steps? How will we move forward? How do we plan for what we do not yet fully understand? I see nurses wearing varying degrees of Personal Protective Equipment (PPE), often lacking essential tools and supplies, trying to safely communicate and manage patients through their entrance into and exit from the hospital.

I see nurses exhausted and depleted. And I realise that cognitive burden is even more of an issue than it was before. The mental and physical burden nurses must carry in this time of need may be life altering for them.

Nurse leaders will face challenges, perhaps unlike any they have faced previously. There will be financial pressures. There will be the business of making sense of a virus we have little understanding of today, but which remains a global threat. And there will be the work of mending those weary from the struggle of the fight. We ought to begin the hard work of looking forward and caring for those who have cared so tirelessly for patients and families.

**Figure 1.**
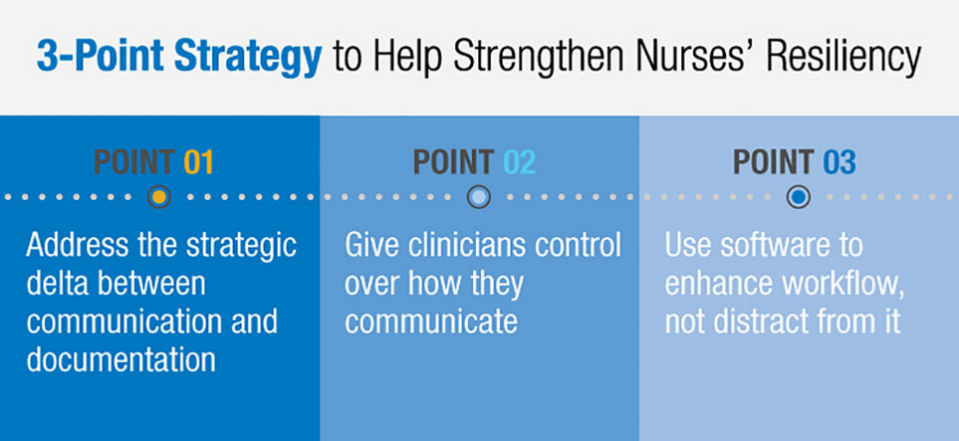
Strategy roadmap of essential steps to aid nurse resiliency.

I will begin the body of this report with a short review of cognitive capacity theory. This is a body of knowledge created by people who have studied the thresholds of the mind’s ability to think, process information, and maintain focus. It offers the foundation for a roadmap for understanding, supporting, and providing essential tools to the people whom we need to help recover (see Figure 1).

## Eight core skills of cognitive capacity

As I wrote in my 2019 CNO Perspective report, cognitive load can be defined as the amount of information a person holds and processes within working memory. Cognitive load often becomes overload for nurses and clinicians because they can rarely control the amount of information that comes to them nor the manner or speed with which it is delivered. Cognitive capacity theory has identified eight core cognitive skills or executive functions^1^:

- Sustained attention: The ability to stay focused on a task for an extended period.
- Response inhibition: The ability to shut out distractions and stay focused.
- Speed of information processing: How quickly someone can process written or spoken information.
- Cognitive flexibility and control: The ability to quickly switch between thoughts.
- Multiple simultaneous attention: The ability to switch attention between activities or to multitask.
- Working memory: The capacity to remember and use relevant information.
- Category formation: The ability to organise information into conceptual categories.
- Pattern recognition: The ability to perceive patterns and anticipate what will happen next.

These cognitive skills often interplay, and leveraging technology designed to streamline clinical workflow can improve a caregiver’s facility with all of them.

Severe cognitive load hinders nurses’ ability to pay attention. It affects the way they respond to the people around them and how quickly they can assimilate information. Cognitive overload often leads to mistakes and medical errors.

My 2019 CNO Perspective report looked at the first four cognitive skills in the list, and this report will focus on the last four (multiple simultaneous attention, working memory, category formation, and pattern recognition). It will explore how these skills play in a strategy for relieving nurses’ cognitive burden and where technology can have the most impact.

### Multiple simultaneous attention

Nothing has brought the challenges of multitasking in the patient care environment into focus like what we have experienced with COVID-19. We have witnessed the unrelenting work of healthcare teams trying to care for patients who can go from a cough to a ventilator in 4 hours.

The constant need to respond in the moment, unable to plan and strategize, impedes nurses’ ability to sustain attention. Nurses need tools and strategies to help them cope with a saturated work environment. Communication technology can help relieve the burden by consolidating information and putting it in the context of the patient.

When managing multiple patients, nurses must commit multiple acts to memory, for example, remembering to change one patient’s intravenous fluid within the hour and to check for another patient’s lab values in 10 minutes. When acts become more complex, mistakes can happen, for example, a nurse focused on a critical ventilated patient might forget to have Arterial Blood Gas (ABGs) drawn on a second patient.

A communication system must be able to send notifications from medical devices with critical information in context of the patient. It must allow the nurse to record reminders and be alerted of critical tasks at designated times. We must begin the hard work of looking forward and caring for those who have cared so tirelessly for patients and families.

Software should allow automated “nurse nudges” to enhance the workflow and support the memory of the very busy nurse.

When care team members synchronise on the same communication software and on technology that supports the rapid delivery of information, the need to multitask is reduced.

### Working memory

Working memory is the ability to remember instruction or keep information in your mind long enough to complete a task. A simple example is researching data for a clinical update document and having to toggle back and forth between the reference and the working document to capture the statement.

As tasks change and information moves in a high-stress, fast-paced patient care environment, multiple demands are placed upon working memory. Working memory becomes taxed beyond its capacity to remember changed values or discreet data points. It is in this space of burdened memory and forgotten tasks that mistakes are often made.

Vocera technology can carry the burden of memory by providing accurate, timely information to relieve clinicians’ cognitive load and help improve patient safety.

#### Category formation

Many clinical leaders reading this have not just seen, but lived, the images of hospitals where COVID-19 patients arrived in endless succession without enough beds or enough equipment. The struggle to assess and categorise inconsistent and changing symptoms can challenge the busy, stressed healthcare team carrying out urgent tasks.

As clinicians, nurses are expected to organise knowledge, to assemble all the lab work, to read all the notes, to look at every relevant detail regarding the patient, and to put the patient status picture together. The nurse then needs to work out how the doctor wants the information communicated. A nurse with four patients might be dealing with eight to 10 or more care team members—specialists and subspecialists, changing throughout a shift.

Vocera communication devices and software streamline the workflow of communication. For example, patients who are on continuous narcotics for pain management may require frequent assessment via a pain score. Having that score readily available to compare over time enables the nurse to understand if the patient’s pain is being relieved. The pain score, vital signs, and medication are all in context of the patient and readily available for the mobile nurse. Our technology can receive and organise information from systems like the Electronic Health Record (EHR) to help relieve the nurse’s burden of forming mental categories and organising information.

#### Pattern recognition

Initially with COVID-19, the symptoms we expected to see were cough, fever, and shortness of breath. Then suddenly, patients started presenting with chest pain, gastrointestinal distress, headaches, and low-grade fever. A busy nurse might receive a patient presenting with gastrointestinal distress and think, “Someone else had that and they tested positive. Who was that? Where did that happen?”

In pattern recognition, the mind matches information from a stimulus with information from a retrieved memory. Communication software that categorises information can relieve the nurse of the burden of pattern recognition.

If a patient presents with an unusual symptom or unusual characteristic, a nurse with the Vocera Vina smartphone app can easily access information about other patients and see their diagnoses, vital signs, and how they presented. The nurse can identify patterns in the information.

## Three-point strategy to help strengthen nurses’ resiliency

Cognitive science provides a foundation for devising a strategy to build nurses’ resiliency. It helps us see how we can support nurses by providing technology that consolidates information in the context of the patient and enables everyone to communicate the same way to reduce cognitive burden. This three-point strategy provides guidance for how these ideas can be applied in the patient care environment.

### POINT 01: Address the strategic delta between documentation and communication

When caregivers are moving through the hospital trying to manage critically ill patients at warp speed and the patients just keep coming, information cannot be logged at a workstation. Care teams are mobile, and information must be too. Cognitive science provides a foundation for devising a strategy to build nurses’ resiliency.

Hospitals rely on the EHR/electronic patient record as a repository of information. If information remains in that repository outside the context of communication, the nurse must continually pair up the EHR and the communication system. This creates another point of cognitive burden, causing the nurse to have to multitask and carry information in memory in order to communicate it.

Documentation and communication are each essential. One does not work without the other, yet today, no single platform can address both. Documentation on the patient resides in the EHR, but for it to be communicated, it must go to an endpoint so the nurse can receive it. A hospital cannot have an EHR without a communication platform and cannot have a communication platform without an EHR. They’re two different things and both are necessary. Documentation and communication must work together to relieve the cognitive burden of clinicians.

### POINT 02: Give clinicians control over how they communicate

The COVID-19 crisis has made it clear that hospitals need to empower nurses to control the way they communicate. Let us say a nurse receives a patient who has been admitted through the ED. He is presenting with shortness of breath. He has a low-grade fever and no headache, but he has gastrointestinal distress. The nurse is thinking, “He needs to be tested for a virus but I’m not really sure, and I do not know whom to notify. Should I notify the infection team who is doing the triage? Should I notify the clinician in the ED who is carrying her own personal phone?”

We must remove the burden of constantly having to adapt to varied preferences. No nurse should have to ask, “Does this doctor want to be paged/bleeped? Does the other one demand I go through the answering service? How do I call the department I need to reach?”

Put everybody—physicians, nurses, and ancillary staff—on the same communication platform. Whether they communicate by texting or calling, everybody needs to be using the same software system in order to communicate efficiently in real time. Select a communication platform that can work for everybody and drive usage behaviour.

Nurses and physicians communicate differently, but they can use the same software. They can use the same nomenclature—the same abbreviations, the same email address format, the same framework—so nurses do not have to work in and out of systems.

### POINT 03: Use software to enhance workflow, not distract from it

The COVID-19 crisis has demonstrated that clinicians need clear, contextual, just-in-time information. There is no time to be searching for information or struggling to communicate. Information must be pushed to the healthcare team on the go. It must be coordinated at the same time, in the same way.

Electronic health record and medical device vendors want to send all their notifications to the nurse who is on the move. If alert and alarm notifications are not actionable, they do not need to go to the nurse. Send only actionable information to a mobile device and lift cognitive burden.

I have listened in on conversations between nurse executives who, during patient surges, are changing and easing documentation requirements. Many are realising they have over-customized software with drop-down boxes to the point where the customisation has caused a degree of cognitive burden because nurses are constantly looking for the proper information. Nurses do not have time to search for critical information when they are under such pressure.

At Vocera, we address the overcustomisation issue by sending only actionable information and by following workflow. We enhance the workflow; we do not add to it. We start by doing assessments to understand a clinical practice. Then we create workflows to support that practice, finding ways to fix what is broken and shore up areas of weakness.

An EHR’s communication applications work best within the documentation side of the EHR. A care team member must be signed into the system to get the message. There is no role-based calling. There are no urgent call teams like stroke or ST-Elevation Myocardial Infarction (STEMI) teams. There is no escalation of communications and notifications.

You cannot communicate in a hospital entirely on a documentation system. It is impossible. Efficient clinical workflow requires purposeful communication and purposeful information. If you need to communicate urgent communication, you must have a communication strategy. If there is a mechanism by which critical information such as lab values, vital signs, and sepsis risk alerts is pushed out from the EHR, that is the communication path.

## Looking forward

Around the world, we’re still sorting through the lessons to be learned. No one has all the answers. What we do know for sure is that we are forever changed. Just like with everything else, we cannot always control what happens, but we can control our response and our preparedness.

How do we ensure we do not run out of supplies or unintentionally spread infectious disease? How do we ensure that communication, which is the very backbone of patient care, is no longer an afterthought or tacked onto another system but is, in fact, a strategy and system of its own?

Communication tools must be part of required PPE. We all have seen nurses with multiple communication devices hanging off their scrubs or in their hands or held near the face. These devices must be cleaned between every patient, which consumes time and supplies. A wearable communication device such as the Vocera Badge that can be worn under PPE allowing for continued communication and receipt of patient information in real time is essential to protect the nurse and facilitate the flow of information.

Some hospitals will continue to manage in crisis mode for some time. Others are seeing a break or slowing in the stream of patients. Recommendations for planning for the next time are starting to surface and financial worries have begun. We do not know for sure what the future holds, including how long we have before the next outbreak or unforeseeable crisis. What we can do is define what is essential, ease the burden when we can, and use the lessons learned to plan for future crises that impact and strain our healthcare system.

Our teams at Vocera are here to help, and I want to hear from you. I welcome you to connect with me on LinkedIn or follow me on Twitter @rhondacno.

- For information about COVID-19 workflow communication solutions, visit www.vocera.com/covid.
- For information about solutions for reducing cognitive overload, visit www.vocera.com/cognitiveoverload.
- To speak with one of our experts about Vocera solutions or to request a demo, email salesweb@vocera.com or call:
  ○ UK: 0800 652 8773
  ○ Canada: 647-490-1637
  ○ Australia: 1800 014-987
  ○ New Zealand: 0800 446 149
  ○ Middle East: 800-0182438

Rhonda Collins, DNP, RN, FAAN, has served as chief nursing officer at Vocera since January 2014. As CNO, she is responsible for working with nursing leadership groups globally to share clinical best practices, help them better understand the value of Vocera solutions, and bring their specific requirements to our product and solutions teams.

Prior to joining Vocera, Rhonda was vice president and business manager for Fresenius Kabi, USA, responsible for the launch of the company’s intravenous infusion pump in the United States. She also led The American Nurse Project, elevating the voice of nurses across the country.

Through her previous experience at Masimo Corporation as vice president of nursing and at Baylor University Medical Center as vice president of women and children’s services, she gained deep experience maximising market share and profitability while building on best clinical and business practices.

Rhonda holds a Doctor of Nursing Practice from Texas Tech University Health Sciences Center and a Master of Science in Nursing Administration from the University of Texas. In 2017 and 2018, Becker’s Hospital Review included her on its “Women in MedTech to Know” and “Female Healthcare IT Leaders to Know” lists. In 2019, she was honoured as one of the 100 most influential women in Silicon Valley, receiving a 2019 Women of Influence Award from the *Silicon Valley Business Journal*.

Rhonda has been a registered nurse for 28 years. She is a frequent speaker on the evolving role of nurses, the importance of communication, and how to use technology to improve clinical workflows and care team collaboration.

### About Vocera

The mission of Vocera is to simplify and improve the lives of healthcare professionals and patients, while enabling hospitals to enhance quality of care and operational efficiency.

In 2000, when the company was founded, we began to forever change the way care teams communicate. Today, Vocera offers the leading platform for clinical communication and workflow. More than 2,100 facilities worldwide, including over 1,700 hospitals and healthcare facilities, have selected our solutions.

Care team members use our solutions to communicate and collaborate with co-workers by securely texting or calling and to be notified of important alerts and alarms. They can choose the right device for their role or task, including smartphones or our hands-free, wearable Vocera Smartbadge and Vocera Badge.

Interoperability between the Vocera Platform and more than 150 clinical and operational systems helps reduce alarm fatigue, speed up staff response times, and improve patient care, safety, and experience.

Vocera (NYSE: VCRA) is publicly traded with the resources and fortitude to help ensure your success with our solutions over the long term. In 2017, Vocera made the list of Forbes 100 Most Trustworthy Companies in America. Learn more at www.vocera.com and follow @VoceraComm on Twitter.
